# Defining functional signatures of dysbiosis in periodontitis progression

**DOI:** 10.1186/s13073-015-0165-z

**Published:** 2015-04-27

**Authors:** Gary P Wang

**Affiliations:** Division of Infectious Diseases and Global Medicine, Department of Medicine, University of Florida College of Medicine, 1600 SW Archer Road, Gainesville, FL 32610 USA; North Florida/South Georgia Veterans Health System, Medical Service, 1601 SW Archer Road, Gainesville, FL 32608 USA

## Abstract

Periodontitis is a common inflammatory disease that leads to tooth loss and has been linked to cardiovascular disease and diabetes mellitus. The periodontal microbiome is highly diverse, and metatranscriptomic studies have indicated that the genes that are expressed by the microbiota are more relevant than the microbial composition in the pathogenesis of periodontitis. A recent study of early metabolic activities in the dysbiotic microbiome reveals a functional signature that distinguishes periodontal sites that will become inflamed, supporting the idea that microbial communities as a whole drive disease progression.

## The periodontal microbiome

Periodontitis is a chronic inflammatory disease that compromises the supporting tissues of the teeth, collectively known as the periodontium, and affects nearly 750 million people worldwide [1]. Periodontitis leads to tooth loss, but emerging research now links periodontitis to a number of systemic diseases, including cardiovascular disease, pre-term delivery and low birth weight, diabetes mellitus, respiratory infections, and osteoporosis. Given the wide-ranging effects of periodontal disease, an understanding of the pathogenic mechanisms that underlie it is crucially important. The current model of periodontal disease progression proposes that changes in periodontal microbiota, or dysbiosis, de-regulate the host immune response, leading to chronic inflammation. We know little, however, about the initiating stage of dysbiosis that leads to disease progression. In *Genome Medicine*, Yost *et al*. [2] report the results of community-wide *in situ* expression analysis in the early stages of dysbiosis associated with periodontitis progression, providing evidence that the entire dysbiotic microbiome drives periodontitis pathogenesis.

It has long been appreciated that changes in periodontal microbiota contribute to periodontitis pathogenesis. A triad of the so-called ‘red complex’, a group of three oral anaerobic bacteria, *Porphyromonas gingivalis*, *Treponema denticola* and *Tannerella forsythia*, has traditionally been considered to cause periodontitis. More recently, advances in high-throughput sequencing technology have revolutionized our ability to examine microbial communities, and it is becoming evident that the real culprit is a dysbiotic microbial community that synergizes to induce chronic inflammation and tissue destruction. The periodontal microbiota is more diverse than previously thought [3-6], with over 700 microorganisms identified as possible components. Many of these are previously unidentified or unrecognized organisms, and up to 100 different taxa can be present at any one site in any given individual [3-5]. This immense diversity of periodontal microbiota, along with the lack of consistent association between any organism or group of organisms and periodontitis, suggests that more organisms need to be considered to delineate fully the pathogenic mechanisms underlying periodontitis.

These sequence-based findings of a diverse, complex periodontal microbiota have led to a new model of periodontal pathogenesis, termed ‘polymicrobial synergy and dysbiosis’ (PSD) [7]. This model proposes that periodontitis is initiated by a dysbiotic microbial community, rather than by select periodontal pathogens, within which different microbial members or specific gene combinations have distinct roles that synergize to shape a microbiota that causes disease. Central to this model is the concept of a synergistic, dysbiotic microbiota that can alter host-microbe homeostasis and facilitate its transition to a chronic inflammatory state. Thus, the whole microbial community drives disease progression.

## Evidence from metatranscriptomics

Two recent metatranscriptomic studies [8,9] have provided support for the idea that the genes expressed by the microbiota and their functions are more relevant to periodontal disease pathogenesis than the actual composition of the microbiota. Jorth *et al*. [9] compared the metatranscriptome of healthy and diseased periodontal sites from patients with aggressive periodontitis. Despite high inter-individual variations in microbial composition, the metabolic gene expression profiles were highly conserved among disease-associated microbial communities. Thus, it appears that there is a high degree of functional redundancy within disease-associated communities, in which different microorganisms with conserved metabolic functions can substitute for each other to cause disease.

In another study, Duran-Pinedo and colleagues [8] compared the subgingival *in situ* genome-wide transcriptome of periodontally healthy and diseased individuals. These investigators found that several functional signatures, including iron acquisition, lipopolysaccharide synthesis and flagellar synthesis, were characteristic of periodontitis. What was surprising was that a large number of virulence factors that were upregulated in diseased microbiomes belonged to organisms not traditionally considered to be periodontal pathogens. In the context of the new PSD model [7], these data suggest that many previously unrecognized bacteria might serve as accessory pathogens that contribute to collectively elevating the virulence of dysbiotic microbial communities, stimulating chronic inflammation and disrupting homeostasis, ultimately leading to periodontal tissue destruction. Nevertheless, the cross-sectional design of this study [8] and that of Jorth *et al*. [9] makes it difficult to determine whether the changes in microbiome composition and metabolic function observed are a cause or a consequence of disease.

## Early metabolic activities in the dysbiotic microbiome

In *Genome Medicine*, Yost *et al*. [2] describe an important contribution to our understanding of the early metabolic activities in the dysbiotic microbiome that lead to periodontal disease progression. Using a combined metagenomic and metatranscriptomic approach, these authors analyzed community-wide microbiome genomes and their gene expression in periodontal sites that progressed over time, compared to sites that remained clinically stable. The overall community composition and their active microbiota were significantly altered in progressive periodontal sites. The overexpression of genes related to cell motility, lipid A and peptidoglycan biosynthesis, and the transport of iron, potassium and amino acids was observed. In addition, several major periodontal pathogens had upregulated expression of a large number of genes and putative virulence factors in sites where disease had progressed compared to the same sites at baseline. By contrast, the microbial composition and *in situ* community-wide gene expression changed only minimally in clinically stable sites from the same subjects.

Several functional signatures were characteristic of progressing sites [2]. At baseline, isoprenoid and polysaccharide biosynthesis, sulfur compound metabolic processes, potassium ion transport and protein kinase C-activating G-protein coupled receptor signaling pathways were highly expressed. When the sites progressed, defined by a 2 mm or greater increase in clinical attachment loss compared to baseline genes associated with pathogenesis, response to oxidative stress, and ferrous iron transport were highly expressed.

Next, when the authors compared baseline samples from progressing and non-progressing sites, they detected notable differences in the overall microbial community and in the components of the active microbiome. In baseline sites that developed disease progression, *P. gingivalis*, several members of the orange complex, including *Prevotella intermedia* and *Eubacterium nodatum*, and species that were previously underappreciated, including *Filifactor alocis*, were found to be more active. Interestingly, the other two members of the red complex, *T. denticola* and *T. forsythia*, were not significantly more active. An increased expression of genes related to proteolysis, sulfur-compound metabolism and response to antibiotics was observed in baseline sites that developed disease progression. The authors indicated that the high activity of *P. gingivalis* at the baseline of progressing sites supports the keystone pathogen hypothesis [10], in which the keystone species (that is, *P. gingivalis*) can elevate the virulence of the entire microbial community, by interacting and communicating with other members of the microbial community to disrupt tissue homeostasis and mediate disease progression.

Interestingly, in the baseline sites that appeared clinically healthy, a large fraction of the microbial community was highly active. This was demonstrated by the overexpression of genes related to citrate, organic ion and lactate transport, sulfur compound metabolic processes, and peptidoglycan catabolism. This finding suggests that in these individuals with periodontitis, clinically healthy sites were already impacted by disease.

## Functional signatures

By comparing gene expression profiles before and after the manifestation of clinical disease, Yost *et al*. [2] were able to define functional signatures that distinguished the early metabolic activities occurring at progressing sites from those at non-progressing sites. These included citrate transport, iron transport, potassium transport, amino-acid transport, isoprenoid biosynthesis and ciliary and flagellar motility. Intriguingly, at baseline, a large number of putative virulence factors were upregulated in a set of organisms not typically associated with periodontitis - S*treptococcus oralis*, *Streptococcus mutans*, *Streptococcus intermedius*, *Streptococcus mitis*, *Veillonella parvula* and *Pseudomonas fluorenscens* - some of which are often associated with periodontal health. Taken together, the findings of Yost *et al*. [2] are consistent with the PSD model [7] and support the idea that whole dysbiotic microbial communities, initially induced by keystone species such as *P. gingivalis*, synergize and drive the progression of periodontitis.

Emerging data have provided insights into the role of dysbiotic microbial communities in periodontitis pathogenesis. Microbial dysbiosis has effects on human physiology that go beyond periodontitis and involve a number of clinically important processes, such as obesity, colitis, inflammatory bowel disease, and colorectal cancer. For example, dysbiotic gut microbiome can lead to a loss of colonization resistance against enteric pathogens and subsequently to colitis caused by *Clostridium difficile*. While the molecular and cellular mechanisms leading to microbial dysbiosis remain poorly understood, this study by Yost *et al*. [2] represents a major step forward in understanding the early microbial activities associated with periodontal disease progression. Importantly, this work contributes additional support for an increasingly appreciated model of polymicrobial synergy and dysbiosis for the pathogenesis of periodontitis. It will lead to a more thorough understanding of this complex disease process, and ultimately to the development of targeted novel therapeutics.

## Abbreviation

PSD: Polymicrobial synergy and dysbiosis
